# Mutagenic Analysis of the Intracellular Portals of the Human 5-HT_3_A Receptor*

**DOI:** 10.1074/jbc.M113.503300

**Published:** 2013-09-12

**Authors:** Jane E. Carland, Michelle A. Cooper, Matthew R. Livesey, Tim G. Hales, John A. Peters, Jeremy J. Lambert

**Affiliations:** From the Division of Neuroscience, Medical Research and Medical Education Institutes, Ninewells Hospital and Medical School, University of Dundee, Dundee DD1 9SY, Scotland, United Kingdom

**Keywords:** Cys-loop Receptors, Neurotransmitter Receptors, Nicotinic Acetylcholine Receptors, Patch Clamp Electrophysiology, Serotonin, Pentameric Ligand-gated Ion Channel

## Abstract

Structural models of Cys-loop receptors based on homology with the *Torpedo marmorata* nicotinic acetylcholine receptor infer the existence of cytoplasmic portals within the conduction pathway framed by helical amphipathic regions (termed membrane-associated (MA) helices) of adjacent intracellular M3-M4 loops. Consistent with these models, two arginine residues (Arg^436^ and Arg^440^) within the MA helix of 5-hydroxytryptamine type 3A (5-HT_3_A) receptors act singularly as rate-limiting determinants of single-channel conductance (γ). However, there is little conservation in primary amino acid sequences across the cytoplasmic loops of Cys-loop receptors, limiting confidence in the fidelity of this particular aspect of the 5-HT_3_A receptor model. We probed the majority of residues within the MA helix of the human 5-HT_3_A subunit using alanine- and arginine-scanning mutagenesis and the substituted cysteine accessibility method to determine their relative influences upon γ. Numerous residues, prominently those at the 435, 436, 439, and 440 positions, were found to markedly influence γ. This approach yielded a functional map of the 5-HT_3_A receptor portals, which agrees well with the homology model.

## Teaser

**Background:** 5-HT_3_R cytoplasmic arginine residues, within a putative amphipathic α-helix (MA helix), influence unitary conductance (γ); how other residues within this motif affect γ is unknown.

**Results:** Alanine-, arginine-, and cysteine-scanning mutagenesis reveal that γ is affected by seven additional residues.

**Conclusion:** The charge of 10 MA helix residues influences γ.

**Significance:** Our findings support a proposed structure of ion-conducting cytoplasmic portals.

## Introduction

The delicate balance between neuronal excitation and inhibition in the central nervous system is crucial to maintaining normal physiological function. The members of the pentameric ligand-gated ion channel (pLGIC)^5^ superfamily are key players in this balance. In eukaryotic organisms, these comprise the excitatory, cation-selective, nicotinic acetylcholine (nACh) (1, 2) and 5-hydroxytryptamine type 3 (5-HT_3_) receptors (3, 4) and the inhibitory, anion-selective, γ-aminobutyric acid type A (GABA_A_) (5, 6) and glycine receptors (7, 8) that are collectively termed the “Cys-loop receptors.” The prokaryotic homologues are pLGICs from *Gloeobacter violaceus* (GLIC) and *Erwinia chrysanthemi* (ELIC) for which, importantly, crystal structures are available (9) complementing that of a eukaryotic glutamate-gated chloride channel (GLC-1, also known as GluClα) (10, 11). The Cys-loop receptors are gated by the binding of their cognate neurotransmitters to permit the transmembrane conduction of selected ions, eliciting either synaptic excitation or inhibition (12, 13).

Cys-loop receptors assemble pseudosymmetrically as either identical, but more commonly homologous, protein subunits that surround a central ion channel pore (13, 14). Each subunit consists of four α-helical transmembrane domains, termed M1 to M4, with the second transmembrane (M2) domain lining the majority of the transmembrane pore. The N and C termini of the subunits are located extracellularly, with the large N-terminal domain harboring the ligand-binding site formed at subunit interfaces, whereas a large intracellular loop extends between the M3 and M4 domains (15).

**FIGURE 1.**
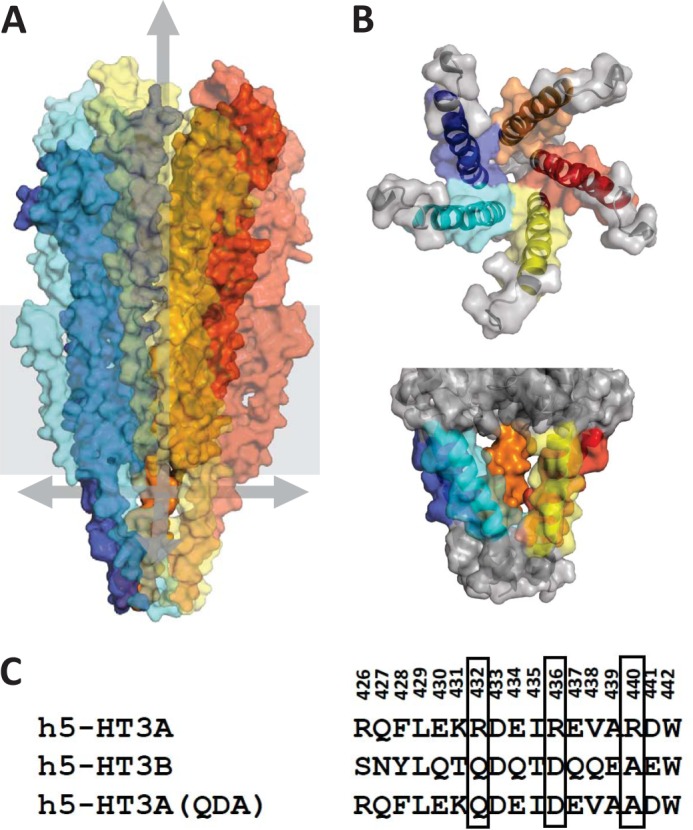
**The putative structure of the 5-HT_3_ receptor ion conduction pathway.** *A*, the complete homology model of the 5-HT_3_A receptor based on the *Torpedo* nACh receptor structure. The pentameric protein is surface-rendered with foreground subunits made transparent. *Arrows* highlight the putative conduction pathway, one pointing to the outer vestibule, the others pointing out three of the five cytoplasmic portals. *B*, five 5-HT_3_A(QDA) receptor MA helices viewed from above (*top panel*) and from the cytoplasm (*bottom panel*). These structures are depicted with both transparent surface rendering and ribbons. The residues substituted by mutagenesis in the current study are indicated in color. Differing colors were used to distinguish each of the five subunits. *C*, the amino acid numbering is that of the human 5-HT_3_A subunit (*h5-HT3A*). The arginine residues that are collectively responsible for the sub-picosiemen single-channel conductance of the human 5-HT_3_A receptor are *boxed* together with the homologous residues within the 5-HT_3_B subunit sequence.

By comparison with the extracellular and membrane spanning domains, the intracellular M3-M4 loop displays the lowest degree of sequence homology across different subunits of the same receptor family or between receptor families and is predicted to be largely unstructured (16–18). Notably, the large intracellular loop is virtually absent from prokaryotic pLGIC subunits (9), and almost complete deletion of this region, among others, was necessary to optimize crystallization of GLC-1, once more suggesting it to be largely unstructured (10). This region of Cys-loop receptors is an established target for phosphorylation (19, 20), and it exerts important influences on, for example, the assembly, maturation, targeting, and gating kinetics of the nACh receptor (18, 21–26). The M3-M4 loop has also been shown to be a critical determinant of single-channel conductance (γ) (27). Cryo-electron microscopic studies of the nACh receptor of *Torpedo marmorata* have revealed the presence of a helical amphipathic stretch, referred to as the membrane-associated (MA) helix, at the C-terminal end of the loop (15) (see Fig. 1). Five such helices extend below the ion channel pore, forming a vestibule that is perforated by five narrow openings, or portals (see Fig. 1). Structure-function studies (28–30) demonstrate that specific residues within human 5-HT_3_A and rat α4β2 nACh receptors positioned within these portals, as inferred by structural models based on homology with the *T. marmorata* nACh receptor, exert a strong influence on γ that is additional to that of the extensively characterized M2 domain and flanking sequences (31, 32). Furthermore, equivalent residues within MA helices of human α1 glycine receptors also influence γ (33). The same region in the 5-HT_3_A receptor additionally impacts upon divalent *versus* monovalent cation permeability, channel gating, and the kinetics of desensitization (34–36). However, ion size selectivity for monovalent cations appears to depend upon the M2 domain (37), and the M3-M4 loop is not essential for receptor function (38).

Structure-function studies of Cys-loop receptors, including the 5-HT_3_A receptor, have extensively probed the M2 domain and flanking sequences for their impact upon ion conduction and selectivity (13, 35, 39–44). The amino acid sequences of the membrane-spanning regions are relatively well conserved across Cys-loop receptors, and systematic mutagenesis essentially confirms the validity of homology models of the 5-HT_3_A receptor M2 domain based on the medium resolution structure (3.6 Å) of the *Torpedo* nACh receptor (see Fig. 1*A*). We previously demonstrated (27, 28) a collective influence on ion conduction of three conserved arginine residues within the MA helix of the 5-HT_3_A receptor (see Fig. 1, *B* and *C*). In particular, the replacement of the three arginine residues by their human 5-HT_3_B subunit counterparts (QDA, respectively, generating a receptor construct coined 5-HT_3_A(QDA)) increased γ 29-fold (27, 28). This influence is interpretable in the context of the portal-like structures observed in cryo-EM studies of the *Torpedo* nACh receptor (see Fig. 1). However, no high resolution structural data are available for the cytoplasmic residues of any of the mammalian Cys-loop receptors, weakening confidence in homology models such as ours (29), based on the *Torpedo* structure.

In this study, we broadened the analysis to include most 5-HT_3_A receptor amino acids within the MA helix lining the putative intrasubunit portals evident in the 5-HT_3_ receptor model (see Fig. 1). There is little conservation of amino acid identity in this region between the 45 human Cys-loop receptor subunits. In the 5-HT_3_A subunit, there are several positively charged residues within the MA helix. By contrast, in nACh receptors, the preponderance of charge is negative (3).

To gain insights into the effect of charge throughout the MA helix, we first established the value for γ of the 5-HT_3_A(QDA) receptor when the uncharged amino acid alanine was present at each position. With these values as a reference, we determined γ when each residue was arginine to examine the influence of positive charge. However, arginine and alanine also differ with regard to their size and hydrophobicity. Therefore, we also introduced a cysteine at each position and attempted methanethiosulfonate (MTS) modification using positively and negatively charged 2-aminoethyl-methanethiosulfonate (MTSEA) and 2-sulfonatoethyl-methanethiosulfonate (MTSES), respectively. This yields additional structural information both providing evidence of the accessibility of each MA helix residue and allowing comparison between the influence of positive and negative charges of equivalent bulk. The alternative approach of scanning the portals with negatively charged amino acids for comparison with the effect of arginine is confounded by the substantially larger volume of the latter. We have previously demonstrated that the volume of residue 436 in the 5-HT_3_A subunit influences γ (29). The influence of multiple residues within the MA helix upon γ strongly supports the existence of cytoplasmic ion conducting portals within the 5-HT_3_A receptor.

## EXPERIMENTAL PROCEDURES

### 

#### 

##### 5-HT_3_A Receptor Constructs and Transfection of Subunit cDNAs

cDNAs encoding human 5-HT_3_A(QDA) and mutant subunit constructs thereof were cloned into prk5. Point mutations were introduced into the 5-HT_3_A construct using standard molecular biological techniques, and all constructs were fully sequenced to confirm fidelity. Wild-type and mutant receptor subunit cDNAs were co-transfected into tsA-201 human embryonic kidney cells with a cDNA encoding green fluorescent protein to identify transfected cells. Transfection was performed by electroporation (400 V, 125 microfarads, infinite resistance) using a Bio-Rad Gene Electropulser II (Bio-Rad). Cells were subcultured twice weekly and incubated in a medium composed of Dulbecco's modified Eagle's medium and 10% (v/v) calf serum, supplemented with 100 μg ml^−1^ streptomycin and 100 units ml^−1^ penicillin. Cells were maintained at 37 °C in an atmosphere of 5% CO_2_ (100% relative humidity). Cell culture reagents were purchased from Life Technologies.

##### Electrophysiology

The outside-out patch configuration was used to record single-channel currents from patches excised from transfected tsA-201 cells. The bath solution contained (in mm): 140 NaCl, 2.8 KCl, 2.0 MgCl_2_, 1.0 CaCl_2_, 10 glucose, and 10 HEPES (pH 7.2 adjusted with NaOH). Patch electrodes were filled with a solution comprising (in mm): 130 potassium gluconate, 5 NaCl, 2 MgCl_2_, 5 EGTA, and 10 HEPES (pH 7.2 adjusted with KOH) and had resistances within the range 4–10 megaohms. 5-HT (10 μm) was dissolved in the bath solution and applied locally by pressure ejection to outside-out patches held at −74 mV (holding potential includes correction for liquid junction potential). Stock solutions of MTS reagents (200 mm) obtained from Toronto Research Chemicals (Ontario, Canada) were stored at −20 °C. Prior to each experiment, MTS reagents were diluted in the electrode solution for outside-out patch experiments to yield a final concentration of 200 μm. Single-channel currents were recorded using an Axopatch 200A amplifier (Axon Instruments) and low pass-filtered at a cut-off frequency of 1 kHz. Data were digitized (Digidata 1322A, Axon Instruments) at 10 kHz onto the hard drive of a PC for subsequent offline analysis. Sections of data recorded from outside-out patches, in which unitary events predominated, were selected for analysis. Multiple Gaussian distributions were fitted (least squares minimization) to all-points amplitude histograms using the Strathclyde Electrophysiology Software developed and provided by J. Dempster (Strathclyde Institute of Pharmacy and Biomedical Sciences, University of Strathclyde, UK). Single-channel conductance (γ) values are reported as the chord conductance determined from the relationship γ = *i*/(*V_m_* − *E*_rev_), in which *i* is the current amplitude of single-channel events, *V_m_* is the holding potential (−74 mV), and *E*_rev_ is the experimentally determined reversal potential. Data were typically obtained from patches subjected to 4–10 challenges with 5-HT. Patches invariably have multiple openings evident during the initial application of 5-HT. Analysis was performed during portions of the recording when unitary events predominated. 5-HT_3_A receptors exhibit inward rectification. However, the relationship between voltage and the conductance of unitary events between −60 and more negative potentials is essentially linear (35).

##### Structural Modeling

The homology model of the 5-HT_3_A receptor was created with the Deepview Swiss-Pdb Viewer using the *T. marmorata* 2BG9 structure, as described previously (28). Images were rendered using PyMOL.

##### Statistics

Data are presented as the arithmetic mean ± S.E. Data sets were compared using one-way analysis of variance with a post hoc Tukey's test. *p* < 0.05 was considered significant.

## RESULTS

The single-channel conductance (γ) of human and mouse homomeric 5-HT_3_A receptors is below the limit of direct resolution by single-channel recording and has been inferred by fluctuation analysis to be within the range 0.3–1.3 pS, dependent, at least in part, upon the concentration of divalent cations within the extracellular medium (27, 28, 34, 45–49). We have previously demonstrated that the replacement of three arginine residues (Arg^432^, Arg^436^, Arg^440^) within the MA stretch of the human 5-HT_3_A subunit by their human 5-HT_3_B subunit counterparts (QDA, respectively) increases γ by 29-fold, as inferred by fluctuation analysis (27) and by 40-fold as revealed directly by single-channel recording (28). To facilitate single-channel analysis of the mutants produced in this study, mutations were introduced into the 5-HT_3_A(R432Q, R436D, R440A) receptor background, henceforth referred to as 5-HT_3_A(QDA). Fig. 1 presents an alignment of residues within the MA stretch of human 5-HT_3_A, 5-HT_3_B, and 5-HT_3_A(QDA) subunits.

### 

#### 

##### Alanine Scan of the Human 5-HT_3_A(QDA) MA Helix

Residues within the human 5-HT_3_A(QDA) MA stretch vary with respect to their physicochemical properties; specifically, 10 are charged (5 positive, 5 negative), 6 are nonpolar, and only 1 is polar (Fig. 1). At the outset of this study, we mutated each residue, from Arg^426^ to Trp^442^, individually to alanine, which presents a simple methyl side chain, thus negating the potential influences of charge and large volume upon γ. Such mutant receptors also provided reference values for γ to which comparisons were made in subsequent amino acid substitution experiments involving the introduction of arginine and cysteine residues and modification of the latter by MTS reagents.

**FIGURE 2.**
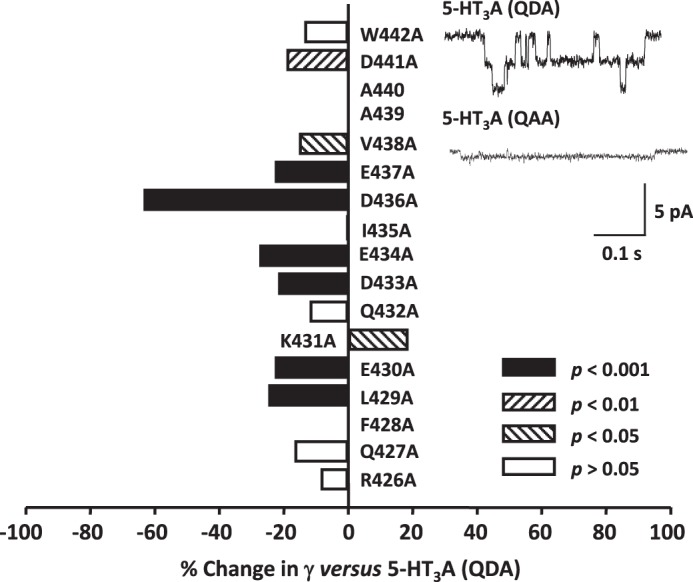
**The influence of alanine substitutions upon the single-channel conductance (γ) of the 5-HT_3_A(QDA) receptor.** The bar graph summarizes the effect of individual replacement of residues 426–438 and 441 and 442 of the 5-HT_3_A(QDA) subunit by alanine. Note that alanine is already present at the 438 and 439 loci of the 5-HT_3_A(QDA) subunit. Data are reported as the mean percentage change in γ caused by each mutation, with the 5-HT_3_A(QDA) receptor acting as control. The mean reference 5-HT_3_A(QDA) receptor γ value was 35.9 pS (Table 1). Statistical analysis was performed by comparing the raw values of γ for the 5-HT_3_A(QDA) and mutant receptor constructs. The *inset traces* are single-channel events recorded from excised outside-out patches expressing either (*top*) 5-HT_3_A(QDA) receptors (control) or (*bottom*) 5-HT_3_A(QAA) receptors (named D436A in the graph).

Within the MA stretch of the human 5-HT_3_A(QDA) subunit construct, alanine occurs naturally at position 439 and replaces the normally resident arginine by mutagenesis at the 440 locus. Expression of the remaining alanine-substituted subunits produced functional receptors, although introduction of the W442A mutation resulted in a very low level of channel activity. It is notable that this tryptophan residue, unlike any of the other amino acids examined in this study, is absolutely conserved across all subunits of cation-selective Cys-loop receptors. In addition, mutation of this residue to alanine in nicotinic α7 receptors severely compromises their cell surface, but not total, expression level (50). The introduction of alanine residues typically resulted in a decrease in the γ of the expressed receptors (Fig. 2, Table 1). With the exceptions of the R426A, Q427A, D432A, and W442A mutations, this change in γ was significant (as indicated in Fig. 2 and Table 1) following the neutralization of charged residues. Thus, for mutations neutralizing negative charge, the percentage of reduction in γ was: E430A, 23%; D433A, 22%; E434A, 28%; D436A, 64%; E437A, 23%; and D441A, 19%. The K431A mutation, removing positive charge, increased γ by 19%. Only two substitutions of polar or nonpolar uncharged residues caused a significant change in γ, those being L429A, which was associated with a 25% reduction, and V438A, which caused a 15% reduction. Of all the exchanges examined, the D436A substitution had the most profound effect upon γ.

**TABLE 1:** **Single channel conductances (γ) for 5-HT_3_A(QDA) receptors mutated at distinct loci to alanine, arginine, or cysteine** Cysteine mutants were challenged to intracellularly applied MTS reagents to determine their accessibility and the influence of charge at specific loci. Data are presented as the arithmetic mean ± S.E. with the number of replications indicated in parentheses.

Locus	Alanine substituent	Arginine substituent	Cysteine substituent		
			Untreated	+MTSEA	+MTSES
	*pS*	*pS*	*pS*		
Arg^426^	32.8 ± 1.4 (7)	35.9 ± 2.0 (5)*^a^*	31.2 ± 1.9 (5)	30.5 ± 1.4 (6)	34.7 ± 1.0 (5)
Gln^427^	29.9 ± 0.6 (5)	34.8 ± 1.2 (5)*^b^*	32.9 ± 1.1 (5)	29.4 ± 1.1 (6)	30.8 ± 1.0 (5)
Phe^428^	35.9 ± 1.4 (7)	39.1 ± 1.2 (5)	35.1 ± 0.4 (5)	27.3 ± 0.6 (6)*^c,d^*	35.6 ± 1.0 (5)
Leu^429^	27.0 ± 0.2 (5)*^e^*	32.6 ± 1.3 (10)*^f^*	27.5 ± 0.9 (8)	24.6 ± 0.4 (5)	29.1 ± 0.7 (5)
Glu^430^	27.7 ± 0.9 (8)*^e^*	30.8 ± 1.6 (7)	30.7 ± 1.1 (5)	28.5 ± 0.5 (6)	39.8 ± 1.4 (7)*^c,d^*
Lys^431^	42.6 ± 0.6 (7)*^g^*	36.6 ± 0.6 (5)*^c^*	42.4 ± 1.6 (5)	38.9 ± 0.4 (5)*^b^*	41.7 ± 0.8 (5)
Gln^432^	31.6 ± 0.7 (6)	24.2 ± 0.7 (7)*^c^*	32.6 ± 1.3 (5)	28.5 ± 0.7 (5)	34.7 ± 1.5 (6)
Asp^433^	28.1 ± 0.7 (5)*^e^*	22.7 ± 0.9 (6)*^b^*	27.2 ± 1.2 (6)	25.3 ± 0.8 (6)	35.5 ± 1.1 (6)*^c,d^*
Glu^434^	26.0 ± 0.4 (7)*^e^*	19.8 ± 0.7 (6)*^c^*	28.0 ± 0.9 (7)	21.4 ± 1.0 (5)*^d,f^*	30.8 ± 0.8 (5)*^f^*
Ile^435^	35.8 ± 0.8 (5)	21.3 ± 0.4 (6)*^c^*	43.7 ± 0.5 (5)*^c^*	19.5 ± 0.8 (5)*^c,d^*	41.5 ± 1.6 (5)*^f^*
Asp^436^	13.1 ± 0.8 (9)*^e^*	6.4 ± 0.2 (3)*^c^*	18.4 ± 0.5 (12)*^c^*	7.2 ± 0.3 (5)*^c,d^*	26.2 ± 1.1 (6)*^c,d^*
Glu^437^	27.7 ± 0.7 (5)*^e^*	21.6 ± 0.6 (5)*^c^*	29.4 ± 0.9 (5)	24.2 ± 1.0 (6)*^h^*	34.7 ± 1.1 (5)*^c,h^*
Val^438^	30.4 ± 1.0 (7)*^g^*	22.7 ± 0.7 (5)*^c^*	31.4 ± 0.8 (6)	23.6 ± 0.3 (5)*^c,d^*	36.1 ± 0.2 (4)*^c,h^*
Ala^439^	35.9 ± 2.0 (5)*^a^*	22.1 ± 1.0 (7)*^c^*	36.3 ± 1.1 (6)	26.9 ± 0.5 (5)*^c,d^*	37.8 ± 0.8 (7)
Ala^440^	35.9 ± 2.0 (5)*^a^*	18.4 ± 0.6 (9)*^c^*	29.2 ± 0.9 (5)*^b^*	17.8 ± 0.9 (5)*^c,d^*	34.4 ± 0.7 (6)*^i^*
Asp^441^	29.0 ± 0.5 (5)*^j^*	27.8 ± 1.2 (3)	32.7 ± 1.2 (6)	29.4 ± 1.3 (5)	35.5 ± 0.9 (5)*^f^*

*^a^* Value for the 5-HT_3_A(QDA) construct lacking additional amino acid substitution.

*^b^ p* < 0.05 when compared with alanine mutant.

*^c^ p* < 0.001 when compared with alanine mutant.

*^d^ p* < 0.001 when compared with untreated cysteine mutant.

*^e^ p* < 0.001 when compared with 5-HT_3_A(QDA) subunit residue.

*^f^ p* < 0.01 when compared with alanine mutant.

*^g^ p* < 0.05 when compared with 5-HT_3_A(QDA) subunit residue.

*^h^ p* < 0.01 when compared with untreated cysteine mutant.

*^i^ p* < 0.05 when compared with untreated cysteine mutant.

*^j^ p* < 0.01 when compared with 5-HT3A(QDA) subunit residue.

##### Arginine Scan of the Human 5-HT_3_A(QDA) MA Helix

**FIGURE 3.**
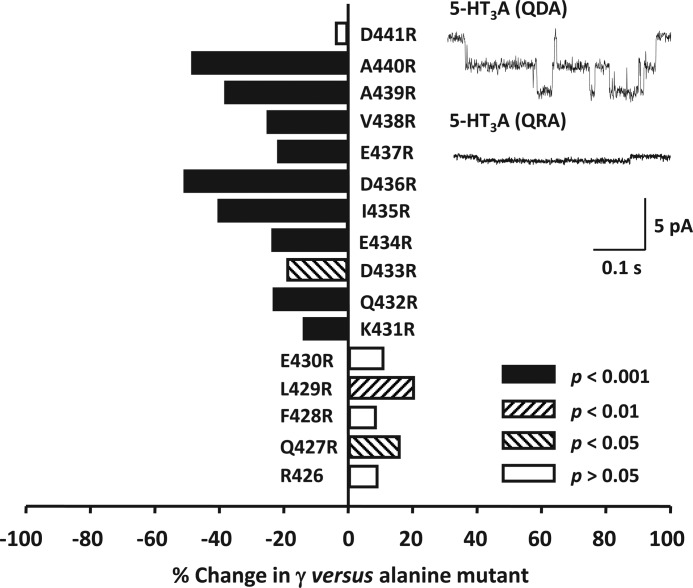
**The influence of arginine substitutions upon the single-channel conductance (γ) of the 5-HT_3_A(QDA) receptor.** The bar graph summarizes the effect of individual replacement of residues 427–441 of the 5-HT_3_A(QDA) subunit by arginine. Note that arginine is already present at the 426 locus of the 5-HT_3_A(QDA) subunit. Data are reported as the mean percentage change in γ caused by each mutation, with the appropriate alanine substituted 5-HT_3_A(QDA) receptor construct acting as the reference. Statistical analysis was performed by comparing the raw values of γ for the arginine- and alanine-substituted 5-HT_3_A(QDA) receptor constructs. The *inset traces* are single-channel events recorded from excised outside-out patches expressing either (*top*) 5-HT_3_A(QDA) receptors (control) or (*bottom*) 5-HT_3_A(QRA) receptors (named D436R in the graph).

Positively charged arginine residues at positions 436 and 440, but not 426 and 432, of the MA helix singularly have a significant impact on the γ of the human 5-HT_3_A receptor (27, 28). In this study, we employed an arginine scan of the 5-HT_3_A(QDA) MA helix to probe for additional locations in which the presence of an arginine residue influences γ. An arginine residue is naturally present at the 426 position of the human 5-HT_3_A(QDA) MA helix. Of the 16 mutant 5-HT_3_A(QDA) receptors produced (from 427 to 442), the W442R mutation resulted in a nonfunctional receptor (Fig. 3, Table 1).

It should be noted that comparisons of γ are between alanine- and arginine-containing receptor constructs. Thus, although we denote mutations as they were actually constructed (*e.g.* L429R), the changes in γ reported are, for example, between Ala^429^ and Arg^429^, not Leu^429^. The sequential introduction of arginine residues from position Lys^431^ to Asp^440^ inclusive resulted in a significant decrease in γ, when compared with alanine controls, at all locations (Fig. 3, Table 1). Inspection of Fig. 3 reveals that the most pronounced decreases in γ occur at the adjacent positions 435 (41%)/436 (51%) and 439 (38%)/440 (49%). Against this overall trend of depression, the Q427R and L429R mutations were associated with a significant increase in γ of 16 and 21%, respectively. Arginine substitution at positions 428, 430, and 441 had no significant effect upon γ (*p* > 0.05). In summary, there is an overall trend for arginine substitutions at positions 427 through to 430 to increase γ and 431 through to 440 to decrease γ.

##### Cysteine Scan of the Human 5-HT_3_A(QDA) MA Helix

**FIGURE 4.**
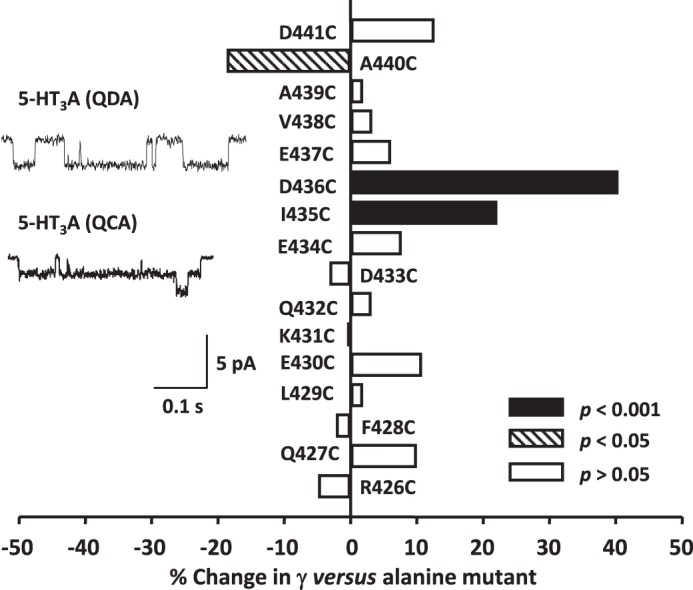
**The influence of cysteine substitutions upon the single-channel conductance (γ) of the 5-HT_3_A(QDA) receptor.** The bar graph summarizes the effect of individual replacement of residues 426–441 of the 5-HT_3_A(QDA) subunit by cysteine. Data are reported as the mean percentage change in γ caused by each mutation, with the appropriate alanine substituted 5-HT_3_A(QDA) receptor construct acting as control. Statistical analysis was performed by comparing the raw values of γ for the cysteine- and alanine-substituted 5-HT_3_A(QDA) receptor constructs. The *inset traces* are single-channel events recorded from excised outside-out patches expressing either (*top*) 5-HT_3_A(QDA) receptors (control) or (*bottom*) 5-HT_3_A(QCA) receptors (named D436C in the graph).

Cysteine residues were introduced sequentially along the MA helix from Arg^426^ to Trp^442^ to enable SCAM analysis. Of the 17 mutant receptors constructed, only the 5-HT_3_A(QDA) W442C construct reduced channel activity to an extent that made it unfeasible to determine the effect of introducing MTS reagents. Typically, the introduction of cysteine residues had little effect on γ when compared with the alanine controls that served to “standardize” the substituted residue (Fig. 4, Table 1). However, a significant increase in γ was caused by the mutations I435C (22%) and D436C (40%), and a decrease (19%) followed the A440C exchange. Overall, there is a good correlation between the effect of alanine and cysteine substitutions upon γ with a tendency for the latter to result in a slightly higher value (Fig. 4). Such an effect may result from the modest negativity of the cysteine side chains, ∼8% of which would be deprotonated at pH 7.2, assuming a p*K_a_* of 8.3. Alternatively, a reduction in hydrophobicity when cysteine substitutes for alanine might underlie the trend to increased γ.

##### Effect of MTS Reagent Application to Human 5-HT_3_A(QDA) MA Helix Cysteine Mutant Receptors

The covalent modification of cysteine residues by MTS reagents allows for the introduction of side chains with differing physico-chemical properties. We have previously shown that that extracellular and intracellular applications of various MTS reagents, including MTSEA and MTSES, to human 5-HT_3_A(QDA) receptors lacking engineered cysteine residues have no effect on γ (29). We examined the effect of positively charged MTSEA and negatively charged MTSES reagents, applied to outside-out patches through the recording electrode on the γ of cysteine mutant receptors. These two reagents were chosen because of their similar molecular volumes. Therefore, a comparison of their effects should not be confounded by differences in steric hindrances caused by the modified cysteine residue (29).

**FIGURE 5.**
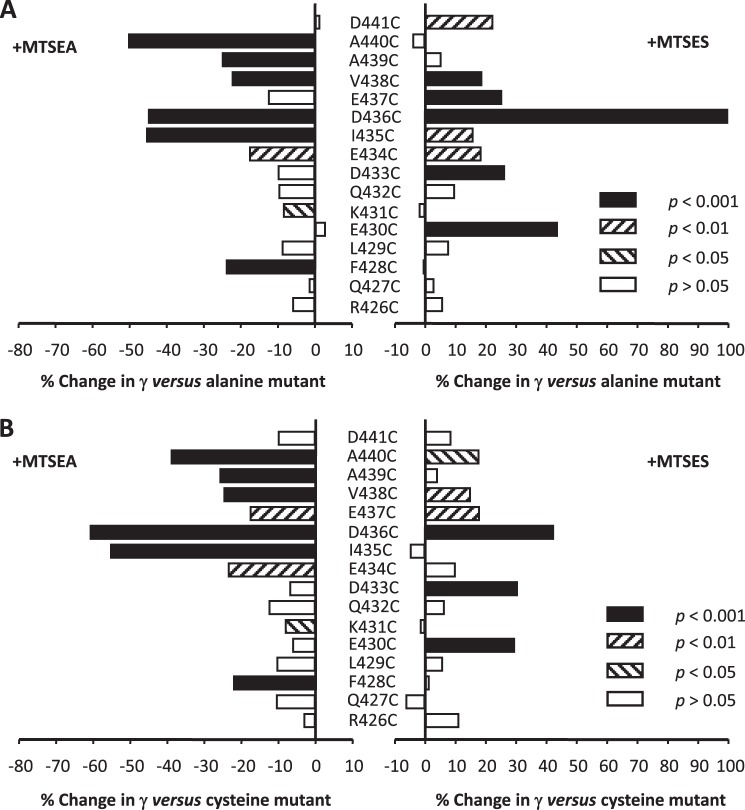
**The influence of modification of engineered cysteine substitutions by MTS reagents upon the single-channel conductance (γ) of the 5-HT_3_A(QDA) receptor.** The bar graphs in *A* and *B* summarize the effect of challenge by either positively charged MTSEA or negatively charged MTSES on the γ the 5-HT_3_A(QDA) receptor in which residues 426–441 had been individually replaced by cysteine. Note that the overall trend is for MTSEA to reduce and MTSES to enhance γ. Data are reported as the mean percentage change in γ caused by the MTS reagents. In *A* and *B*, the appropriate alanine- or cysteine-substituted 5-HT_3_A(QDA) receptor constructs, respectively, serve as the reference. Statistical analysis was performed by comparing the raw values of γ.

**FIGURE 6.**
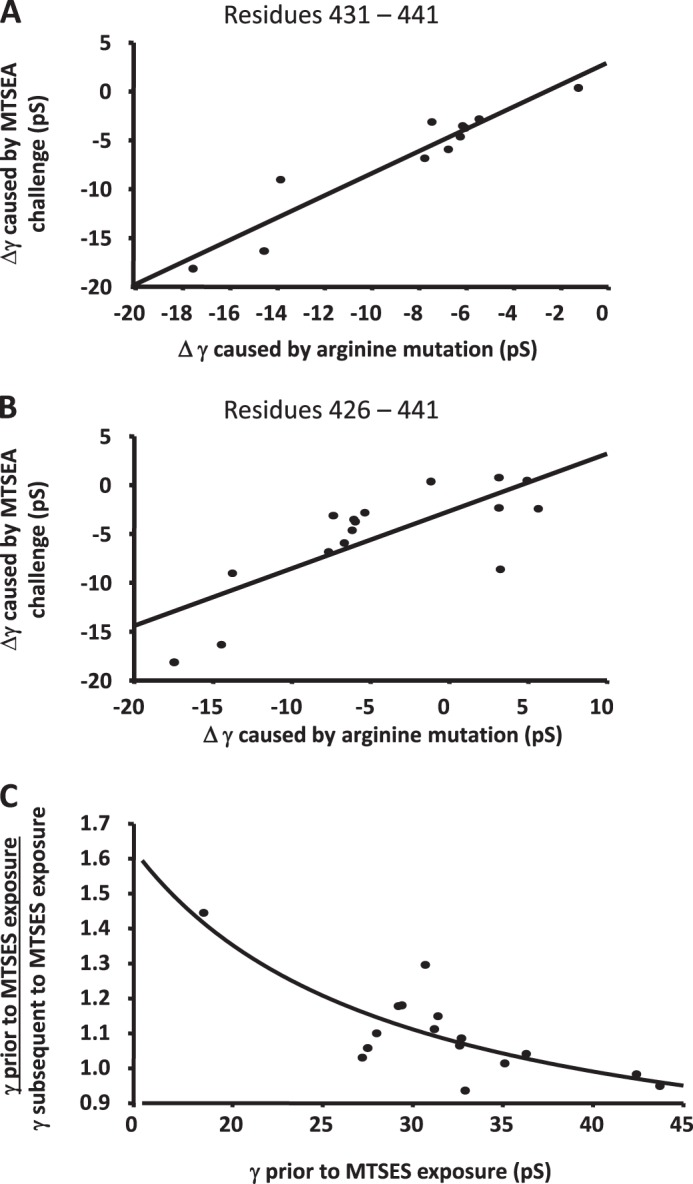
**The influences of arginine substitution and modification of engineered cysteine substitution by MTS reagents upon the single-channel conductance (γ) of the 5-HT_3_A(QDA) receptor.** *A*, plot depicting the strong correlation (*r*^2^ = 0.89) between the change in γ produced by arginine substitution and by the reaction of substituted cysteine residues by positively charged MTSEA at residues 431–441, inclusive, both compared with alanine. *B*, inclusion of residues 426–430 at which the presence of arginine was associated with an increase in conductance *versus* alanine controls weakens the correlation (*r*^2^ = 0.58). *C*, correlation (*r*^2^ = 0.68) between the magnitude of the increase in γ produced by challenge with MTSES *versus* the γ of the construct without exposure to the MTS reagent.

For hydrophilic MTS reagents to react with a substituted cysteine residue, the latter must reside in an accessible aqueous environment (51). The positively charged aminoethyl moiety donated to a cysteine residue by MTSEA results in a side chain with a volume and charge similar to that of an arginine residue, although the latter is considerably more basic. Nevertheless, qualitative similarities between the effects of arginine substitutions and covalent modification of cysteine residues by MTSEA would be anticipated for those cysteine residues that are accessible. Application of MTSEA (200 μm) within the patch pipette typically resulted in a decreased γ when compared with the matched alanine controls. The suppression of γ was significant for receptors containing the F428C, K431C, E434C, I435C, D436C, V438C, A439C, and A440C mutations, the greatest decrease being associated with modification of cysteine residues at positions 435 (46%), 436 (45%), and 440 (50%) (Fig. 5*A*, Table 1). However, such a comparison includes the effect upon γ of the cysteine substitution itself, which in three instances (see above) was significant. Thus, we also analyzed the effect of MTSEA application employing the γ of unmodified cysteine constructs as a reference to isolate the change in γ specifically due to the reagent (Fig. 5*B*). Using this criterion, MTSEA caused significant reductions in γ at positions 434 through 440 inclusive, in addition to 428 and 431. Notably, the introduction of arginine residues at positions 431 through 440 also caused significant reductions in γ in comparison with alanine controls (see above). At all other loci, MTSEA treatment was associated with a trend toward a reduction in γ. Moreover, the effects upon γ of arginine substitution and challenge with MTSEA at positions 431 through 441 were in excellent quantitative agreement (*r*^2^ = 0.89), the data being fitted by a line of regression with a slope (1.13) close to unity (Fig. 6*A*). However, although arginine substitution at residues 426 through 430 tended to increase γ in comparison with the alanine controls, MTSEA tended to produce the opposite effect. Inclusion of the data obtained for these residues resulted in a much poorer correlation between the effect of arginine substitution or treatment with MTSEA upon γ (*r*^2^ = 0.58; Fig. 6*B*).

The application of MTSES to cysteine mutant receptors to potentially donate the negatively charged ethylsulfonate moiety tended to increase γ when compared with the appropriate alanine controls at most loci examined. Despite the substantially increased side chain volume of the covalently modified cysteine residues *versus* alanine, MTSES never caused a significant decrease in γ. Indeed, significant increases in γ in comparison with alanine controls occurred at mutants E430C, D433C, E434C, I435C, D436C, E437C, V438C, and D441C (Fig. 5*A*, Table 1). When the data were analyzed employing the γ of unmodified cysteine constructs as a reference, significant increases in γ were found at fewer loci (*i.e.* 430, 433, 436, 437, 438, and 440; Fig. 5*B*, Table 1). In this regard, it should be recalled that the introduction of cysteine itself at positions 435 and 436 significantly increased γ in comparison with alanine controls and tended toward an increase at several other positions (Fig. 4). We therefore examined whether there is a relationship between the γ of the cysteine mutants before and following the application of MTSES, suspecting that the maximal observable effect of residue modification might be limited by a barrier to permeation elsewhere within the conduction pathway. Fig. 6*C* indicates that a correlation (*r*^2^ = 0.68) exists between γ prior to and after MTSES application such that the largest percentage increases tend to be registered for mutants with a lower γ prior to MTSES treatment. By contrast, there was no correlation between the percentage decrease in γ caused by MTSEA and the γ of the cysteine mutant receptors (data not shown).

It may not be coincidental that the maximum value for γ found in this study (43.7 pS) is remarkably close to that of the human 5-HT_3_A receptor in which the entire intracellular loop was replaced by the heptapeptide linking the M3 and M4 domains of the pLGIC GLIC from the prokaryote *Gloeobacter violaceus* (*i.e.* 43.5 pS (38)).

## DISCUSSION

In this study, by using a combination of alanine- and arginine-scanning mutagenesis, SCAM, and single-channel recording from outside-out membrane patches, we have identified residues within the intracellular MA helix of the human 5-HT_3_A receptor that influence ion permeation. SCAM and site-directed mutagenesis have previously been applied to the mouse 5-HT_3_A receptor to infer or identify the residues within the M2 domain and flanking sequences that line the ion channel and influence ion selectivity (41–43). The present study utilizes a similar strategy but with the considerable advantage that changes in single-channel, rather than macroscopic, current amplitude were determined, obviating ambiguities in interpretation that might arise from perturbations in receptor kinetics (*e.g.* Ref. 41). We have previously demonstrated the dynamic modification of a cysteine residue introduced at the 436 locus by MTS reagents (29), and in this study, a further 15 loci were probed, representing the first comprehensive evaluation of the influence of the MA stretch as a whole upon γ.

The following criteria may be applied to infer that a particular residue within the MA stretch is a component of the conduction pathway: (i) replacement of a negatively or positively charged residue by alanine within the 5-HT_3_A(QDA) receptor construct decreases or increases γ, respectively; (ii) the introduction of an arginine residue decreases γ when compared with the appropriate alanine-substituted control; (iii) reaction of an engineered cysteine residue with MTSEA or MTSES decreases or increases γ, respectively, when compared with alanine- or cysteine-substituted controls; and (iv) the introduction of an arginine residue and the reaction of an engineered cysteine residue with MTSEA produce a qualitatively similar effect upon γ. Criteria iii and iv can only be satisfied if the engineered cysteine residue resides within an aqueous environment and is thus accessible for covalent modification by hydrophilic MTS reagents (51). Moreover, if the introduction of an arginine residue at a particular locus has no significant effect upon γ when compared with the appropriate alanine control, a lack of an observable effect of MTSEA (or MTSES) upon the γ of an engineered cysteine mutant clearly does not imply that residue to be inaccessible to the MTS reagent; instead such an outcome is consistent with the residue lying outside the conduction pathway.

**FIGURE 7.**
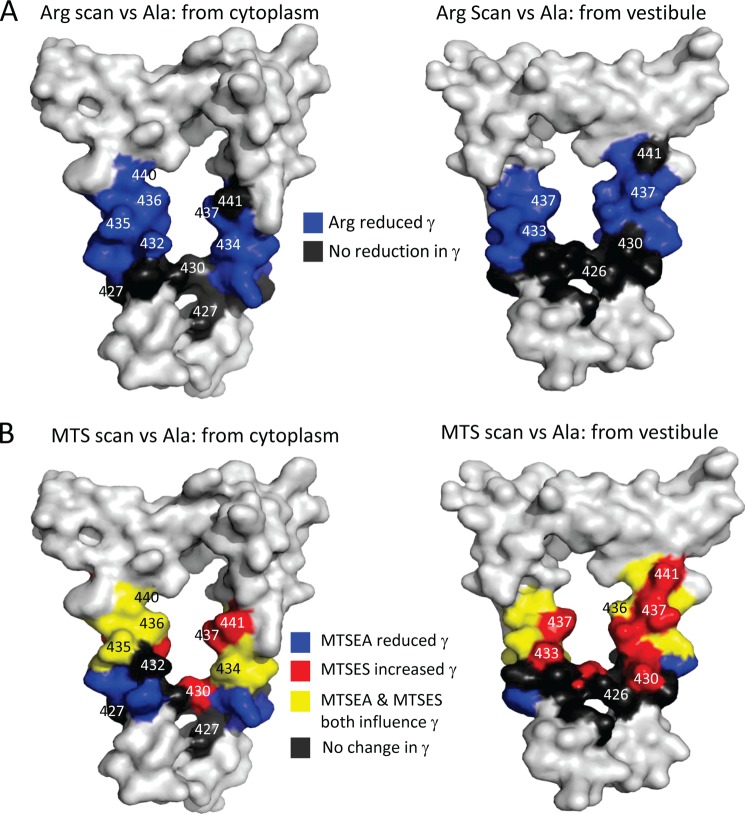
**Residues that determine single-channel conductance (γ) mapped onto a model of a 5-HT_3_A receptor cytoplasmic portal.** *A*, surface renderings of an homology model of a 5-HT_3_A receptor portal formed by adjacent MA helices, viewed from the cytoplasm (*left panel*) or from within the inner vestibule (*right panel*). When arginine residues were present at the positions rendered in *blue*, 5-HT_3_A receptors exhibited significantly reduced γ values when compared with their alanine equivalents (Fig. 3). When arginine residues were present at the positions rendered in *black*, the γ values of 5-HT_3_A receptors were either unchanged or slightly elevated when compared with their alanine equivalents. *B*, the same surface renderings of MA stretches represented in *A*, colored according to the effect of the MTS reagents, MTSEA and MTSES, on 5-HT_3_A receptors into which cysteine residues were substituted at the positions indicated. Cysteine substituents rendered in *yellow* were associated with significantly decreased and increased γ upon MTSEA and MTSES treatment, respectively, when compared with their alanine equivalents (Fig. 5*A*). Those indicated in *blue* or *red* only exhibited a decrease by MTSEA or an increase by MTSES, respectively, when compared with their alanine equivalents. Cysteine substituents rendered in *black* were unaffected by both MTSEA and MTSES when compared with their alanine equivalents. The 5-HT_3_A receptor was modeled on the *T. marmorata* structure (see “Experimental Procedures”).

On the basis of the 4-Å model of the intracellular vestibule of the *Torpedo* nACh receptor (15, 52) and the homology model of the human 5-HT_3_A receptor derived from that structure (29) (Fig. 7), we anticipated that a potentially large number of residues within the MA stretch might impact upon γ, in addition to the 436 and 440 loci identified in previous studies (27–29). Such a suggestion arises from the fact that numerous residues of each MA stretch helix are predicted to face into the centrally located cytoplasmic vestibule or the lateral windows that are framed by adjacent helices (Fig. 7). It should be noted that adjacent helices are rotated (by 72° in a perfectly symmetrical pentamer) with respect to each other, thus placing different residues from each of the two MA helical frames within the lateral window. In addition, the MA stretch may be a mobile structure, as suggested by changes in the conformation of the large intracellular loop of the mouse 5-HT_3_A receptor subsequent to agonist binding (53).

Confining initial consideration to those loci at which arginine substitution caused a significant decrease in γ when compared with the alanine matched controls (*i.e.* residues 431–440 inclusive, rendered in *blue* in Fig. 7*A*), MTSEA also caused a significant decrement in the γ of receptors engineered to express a cysteine residue at 7 of these 10 positions (431, 434, 435, 436, 438, 439, and 440, rendered in *blue* or *yellow* in Fig. 7*B*) when also compared with alanine controls. Essentially the same pattern emerged when the comparison was made with the unmodified substituted cysteine serving as control, with the exception that residue 437 additionally displayed a significant decrease in γ. For those substituted cysteine residues that did not yield a significant reduction in γ following treatment with MTSEA (*i.e.* the 432 and 433 loci), the trend was nonetheless toward decreased conduction. In addition, constructs in which the negatively charged residues within the 432–440 sequence of the 5-HT_3_A(QDA) receptor were mutated to alanine displayed (with the exception of Asp^432^) a significant reduction in γ, whereas the mutation of the solitary positive charge in this region (*i.e.* K431A) resulted in augmented γ. Thus, the effects of introducing positive charge (via either arginine substitution or covalent modification by MTSEA) or neutralization of negative charge (by mutation to alanine) are remarkably consistent across the 431–440 region.

Additional evidence for the involvement of the residues 433–438 inclusive in ion conduction was provided by a significant potentiation, when compared with alanine controls, of the γ of the cysteine mutant constructs as a result of challenge with MTSES. When compared with the unmodified substituted cysteine as control, MTSES treatment was associated with a significant increase in γ at the 433, 436, 437, 438, and 440, but not the 434 and 435 loci. We directly compared the γ of the cysteine engineered constructs that had been challenged with MTSEA or MTSES. As is clear from inspection of Fig. 5, the two treatments generally had opposite influences upon γ over the loci 432–441 (rendered in *yellow* in Fig. 7*B*), and at all of these, the difference in γ was significant. However, in specific instances, MTSEA significantly reduced γ, whereas MTSES did not affect this parameter in comparison with the cysteine control (*i.e.* at the 431, 434, 435, and 439 loci, rendered in *blue* in Fig. 7*B*). As mentioned above, a lack of effect of MTSES does not necessarily preclude the contribution of a residue to the permeation pathway because other components of the ion pore may exert a rate-limiting influence upon γ, or the increase in side chain volume may negate any influence of introducing negative charge. Conversely, MTSES elevated γ at residues 430 and 433, but MTSEA did not cause a significant reduction (rendered in *red* in Fig. 7*B*). It is possible that the environment local to residues 430 and 443 favors deprotonation of the primary amino group of MTSEA (p*K_a_* = 8.5 (51)), rendering the compound neutral. Alternatively, anionic MTSES may have greater access to the residues 430 and 433 than cationic/neutral MTSEA. Irrespective of such complications, arginine introduced at loci 431–440 inclusive decreased γ, and cysteine present at all of these positions, except 432, reacted with either MTSEA or MTSES. We thus conclude that all of the residues, with the possible exception of position 432, are in an aqueous environment and influence γ, albeit to a variable extent.

The effects of arginine substitution or covalent modification of engineered cysteine residues by MTS reagents are most profound at the 435, 436, 439, and 440 loci (Fig. 7). In an α-helical structure, residues 435 and 440 would subtend an angle of 140°, and we tentatively suggest that it is this arc of the helix that most closely impinges upon the permeation pathway.

At positions 426–430 inclusive, the introduction of an arginine residue either had no significant effect upon or caused an increase in γ when compared with the 5-HT_3_A(QDA) receptor construct. Neither effect is compatible with these residues contributing to the permeation pathway, consistent with the structural model in which these residues are below the bottom of the portal (rendered in *gray* in Fig. 7*A*). However, such an interpretation is complicated by the observation that replacement of Leu^429^ and Glu^430^ by alanine caused a significant reduction in γ and that the mutation Q427C caused a small, but significant, increase. In addition, MTSEA decreased the γ of receptors containing a cysteine residue and position 428, whereas MTSES increased the γ of receptors containing a cysteine at 430. However, MTSES and MTSEA did not have opposing effects on either of these receptor constructs. The general failure of residues within the 426–431 positions to exhibit consistent changes in γ with modification of charge, either through mutagenesis or SCAM, suggests that these residues lie outside the sphere of influence of the portal. Collectively, these findings suggest that position 430 is below the bottom of the portal, in agreement with the structural model.

The sphere of influence of portal residue charge on γ extends toward the upper aspect of the portal structure as far as position 440. Residue 441 responded inconsistently to charge modification, suggesting that it lies at the upper limit of the portal structure in keeping with the model. Proceeding beyond this position with arginine or cysteine scanning to the entirely conserved tryptophan at 442 had detrimental effects most likely upon gating by analogy to the α7 nicotinic nACh receptor (50).

An aspect of the 5-HT_3_A receptor portal structure that remains largely untested are the positions of the residues spanning its “ceiling.” The model predicts that these residues are contributed by the beginning of the M3-M4 loop and the cytoplasmic M1-M2 loop. Additional systematic mutagenesis will be required in future studies to probe this aspect of the homology model. Nonetheless, our results indicated that the residue conventionally denoted -4′ (*i.e.* the cytoplasmic ring (31)) when mutated from asparagine (in the human 5-HT_3_A receptor) to aspartate (in the murine 5-HT_3_A receptor) increases γ by ∼10 pS in the context of the human 5-HT_3_A(QDA) receptor construct.^6^

More challenging will be progress toward determining the structure of the M3-M4 loop of Cys-loop receptors lying outside the *Torpedo* nACh receptor model. Complete and higher resolution structural models of GLIC and ELIC are of no help in this regard because these bacterial pLGICs lack the large intracellular loop that is a hallmark of Cys-loop pLGICs and the crystallization of GLC-1 was achieved with the loop largely deleted (10). The missing link between the beginning of the M3-M4 loop and the start of the MA helix is a stretch of 85 amino acids in the human 5-HT_3_A subunit for which there is no structural information. We recently probed this region by deleting stretches of amino acid residues of variable length from close to the start of the MA stretch progressing in the N-terminal direction toward M3 (30). The analysis revealed that deletions that truncated the loop length to between 90 and 75 amino acids were well tolerated using inward rectification of macroscopic current responses to 5-HT as a convenient index of the influence of the MA stretch (30). However, truncation to 70 residues abolished rectification and also increased γ. Interestingly, the M3-M4 loop of all human Cys-loop receptor subunits exceeds 70 residues, suggesting that this is the minimum length necessary to maintain unperturbed function (30).

Our developing functional map of the 5-HT_3_A receptor (27, 29, 30, 35) reveals that, despite the relatively poor conservation of amino acids within MA helices, residues that obey criteria for inclusion as components within the conduction pathway lie within the portal structure derived from the *Torpedo* model.

## Footnotes

6 M. R. Livesey, J. J. Lambert, and J. A. Peters, unpublished data.

## Footnotes

5 The abbreviations used are:

pLGIC: pentameric ligand-gated ion channel
nACh: nicotinic acetylcholine
5-HT_3_: 5-hydroxytryptamine type 3
MA: membrane-associated
MTS: methanethiosulfonate
MTSES: 2-sulfonatoethyl-methanethiosulfonate
MTSEA: aminoethyl-methanethiosulfonate
SCAM: substituted cysteine accessibility method
pS: picosiemens.
